# Whole-Genome Sequence of Streptococcus iniae Strain AH1, Isolated from Hybrid Tilapia (Oreochromis niloticus × Oreochromis aureus)

**DOI:** 10.1128/mra.00087-22

**Published:** 2022-05-26

**Authors:** Ahmed H. Al-Harbi

**Affiliations:** a Life Science and Environment Research Institute, King Abdulaziz City for Science and Technology, Riyadh, Saudi Arabia; University of Rochester School of Medicine and Dentistry

## Abstract

Here, we describe the whole-genome sequence of Streptococcus iniae strain AH1, which was isolated from moribund farmed hybrid tilapia (Oreochromis niloticus × Oreochromis aureus) in Saudi Arabia. The genome is composed of a single linear chromosome of 2,068,661 bp, with a G+C content of 36.8%.

## ANNOUNCEMENT

Streptococcus iniae is a hemolytic, non-Lancefield-classified Gram-positive pathogenic bacterium that was isolated originally from a subcutaneous abscess of a captive Amazon freshwater dolphin, Inia geoffrensis, in 1976 (1). *S*. *iniae* affects numerous freshwater and marine fish species, including tilapia, causing significant economic losses in the aquaculture industry worldwide (2–4). In addition, *S*. *iniae* is also known as an opportunistic zoonotic pathogen, causing serious infectious disease in humans (5–8).

*S*. *iniae* AH1 was isolated from moribund farmed hybrid tilapia (Oreochromis niloticus × Oreochromis aureus) in March 1992 in Saudi Arabia as described previously (9, 10).

A single colony of *S*. *iniae* AH1 was grown aerobically overnight in tryptone soya broth (Oxoid), at 30°C. Genomic DNA was extracted using the DNeasy blood and tissue kit (Qiagen), following the manufacturer’s instructions. DNA quantity and quality were checked with a NanoDrop spectrophotometer (Thermo Fisher Scientific) and gel electrophoresis, respectively. The whole genome was sequenced using a combination of the PacBio Sequel II platform (Pacific Biosciences, USA) and the NovaSeq 6000 platform (150-bp paired-end reads) (Illumina, USA). For PacBio sequencing, genomic DNA was sheared using the g-TUBEs (Covaris, Woburn, MA) and then size selected to greater than 10 kb using BluePippin, end repaired and ligated with universal hairpin adapters using the SMRTbell template prepare kit v1.0 (Pacific Biosciences) according to the manufacturer’s instructions, and sequenced using PacBio P6-C4 chemistry and 240-min movies. The Illumina sequencing library was prepared using the Nextera XT library preparation kit (Illumina) following the manufacturer’s instructions.

The reads were adapter trimmed using Trimmomatic 0.30 with a sliding window quality cutoff of Q15 (11). The library quality was analyzed by Qubit and quantitative PCR (qPCR), and the average fragment size was estimated using an Agilent (Santa Clara, CA, USA) 2100 bioanalyzer. PacBio sequencing yielded a total of 184,455 subreads (mean subread length, 12,636 bp) with an *N*_50_ value of 14,277 bp, totaling 2.33 Gbp. *De novo* assembly was performed using Flye v2.8 (12) and Mecat2 vJAN-2020 software. A total of 7,892,050 raw Illumina paired-end reads were generated, and 7,761,264 clean reads were used for error correction with Pilon v1.22 (13) for the final genome assembly. The quality and completeness of the assembled genome were assessed by benchmarking universal single-copy orthologs (BUSCO) (v4.1.4) (14) with the bacteria_odb10 data set (created 6 March 2020). This resulted in 95.2% complete genes, with 95.2% of the genome with single copy genes, 0.0% duplicated genes, 0.8% fragmented genes, and 4.0% genes missing. The average genome coverage was 938.54×. The genomes were annotated by NCBI using the Prokaryotic Genome Annotation Pipeline (PGAP) v4.2 (15). Default parameters were used for all software unless otherwise specified.

The whole-genome sequence of *S. iniae* AH1 consists of 5 contigs ranging from 1,143 bp to 2,068,661 bp, which had a combined length of 2,178,034 bp; with a G+C content of 37.3%; and with 1,956 protein-coding sequences (CDSs), 68 tRNAs, and 18 rRNAs (Table 1).

**TABLE 1:** Main genome features of Streptococcus iniae AH1

Genetic element	Genome size (bp)	G+C content (%)	Topology	No. of coding sequences	No. of rRNAs	No. of tRNAs	No. of proteins	No. of pseudogenes
Chromosome	2,068,661	36.8	Linear	1,951	18	68	1,864	53
Contig 2	4,202	0	Linear	1	0	0	0	0
Contig 4	1,143	0	Linear	1	0	0	1	0
Contig 6	97,500	52.2	Linear	2	0	0	1	1
Contig 7	6,528	6.1	Linear	1	0	0	1	0
Total or average^*a*^	2,178,034	37.3		1956	18	68	1,867	54

a An average value for G+C content (%) is indicated.

The resulting chromosome was 2,068,661 bp, with a G+C content of 36.8%, and was predicted to carry 1,951 protein-coding sequences (CDSs), 68 tRNAs, and 18 rRNAs, which is consistent with the other *S. iniae* genomovar strains sequenced previously, namely, *S. iniae* 89353 (16) and *S. iniae* YM011 (17).

### Data availability.

The whole-genome sequence of *S. iniae* strain AH1, reported in the manuscript, has been deposited at DDBJ/ENA/GenBank under the accession number JAHZUZ000000000, BioProject accession number PRJNA742166, and BioSample accession number SAMN19929365. The version described in this paper is the first version, JAHZUZ010000000. The raw Illumina and PacBio sequence reads were deposited in the Sequence Read Archive (SRA) under accession number SRR17134084 and SRR17157250, respectively.
